# Virtual Plants Need Water Too: Functional-Structural Root System Models in the Context of Drought Tolerance Breeding

**DOI:** 10.3389/fpls.2017.01577

**Published:** 2017-09-26

**Authors:** Adama Ndour, Vincent Vadez, Christophe Pradal, Mikaël Lucas

**Affiliations:** ^1^Laboratoire Mixte International Adaptation des Plantes et Microorganismes Associés Aux Stress Environnementaux (LAPSE), Dakar, Senegal; ^2^Laboratoire Commun de Microbiologie (IRD-ISRA-UCAD), Dakar, Senegal; ^3^CERES, IRD, Université de Montpellier, UMR DIADE, Montpellier, France; ^4^Département Maths/Informatique, Faculté des Sciences et Techniques, Université Cheikh Anta Diop, Dakar, Senegal; ^5^International Crops Research Institute for the Semi-Arid Tropics (ICRISAT), Patancheru, India; ^6^UMR AGAP, Univiversité de Montpellier, CIRAD, INRA, Inria, Montpellier SupAgro, Montpellier, France

**Keywords:** functional structural plant model, drought, phenotyping, root system architecture, plant development and physiology, breeding

## Abstract

Developing a sustainable agricultural model is one of the great challenges of the coming years. The agricultural practices inherited from the Green Revolution of the 1960s show their limits today, and new paradigms need to be explored to counter rising issues such as the multiplication of climate-change related drought episodes. Two such new paradigms are the use of functional-structural plant models to complement and rationalize breeding approaches and a renewed focus on root systems as untapped sources of plant amelioration. Since the late 1980s, numerous functional and structural models of root systems were developed and used to investigate the properties of root systems in soil or lab-conditions. In this review, we focus on the conception and use of such root models in the broader context of research on root-driven drought tolerance, on the basis of root system architecture (RSA) phenotyping. Such models result from the integration of architectural, physiological and environmental data. Here, we consider the different phenotyping techniques allowing for root architectural and physiological study and their limits. We discuss how QTL and breeding studies support the manipulation of RSA as a way to improve drought resistance. We then go over the integration of the generated data within architectural models, how those architectural models can be coupled with functional hydraulic models, and how functional parameters can be measured to feed those models. We then consider the assessment and validation of those hydraulic models through confrontation of simulations to experimentations. Finally, we discuss the up and coming challenges facing root systems functional-structural modeling approaches in the context of breeding.

## Introduction

At the advent of the Green Revolution in the 1960s, the world population was numbered at 3 billion people. Roughly 50 years later, it reached 7 billion. According to the 2015 revised world population prospects of the United Nations, median estimates place world population at almost 10 billion by the year 2050 (esa.un.org; Lee, 2011). Feeding the current and coming world is a key challenge, and is strongly conditioned by the possibility of extending the practices of the Green Revolution in developing world regions such as Sub-Saharian Africa. Agricultural production worldwide is facing rising multifactorial problems among which are increasing pressure on arable lands, decreasing soil qualities, rising cost of fertilizers and energy, and climatic change. Regarding this last point alone, it is expected that changes in meteorological pattern (precipitation and temperature) will result in decreasing the mean yields of all crops (Knox et al., 2012) especially due to drought, one of the mains constraints for crop productivity (Lynch et al., 2014). This in turn will adversely impact food security in regions where the bulk of the population is coping with chronic hunger and malnutrition (Schmidhuber and Tubiello, 2007; Lobell et al., 2008; Lobell and Gourdji, 2012).

One way for breeders to deal with emerging drought episodes is to create cultivars with improved drought tolerance. This is particularly critical for the subsistence crops used in developing countries where people are almost completely reliant on the crop effective adaptive capacity for their sustenance (Sultan et al., 2013). One particular target of interest in this context is the plant root system. Roots are the organs responsible for the uptake of nutrients and water in the soil. Their efficiency depends on several factors, the main one being their spatial organization within the soil, or root system architecture (RSA) (Den Herder et al., 2010; Draye et al., 2010). The RSA is the result of the interaction between the genetic programming of developing roots and their response to a specific growth environment (Orman-Ligeza et al., 2014). Consequently, RSA developmental plasticity is of major importance as it determines the plant adaptability to environmental constraints such as those of drought-subjected environments (Tuberosa et al., 2002a,b; Lynch, 2007; Draye et al., 2010; Tardieu, 2012; Leitner et al., 2014b). Another important aspect of roots that has been largely overlooked is their functionality and in particular hydraulic processes that facilitate water transport and may explain a number of critical phenotypes, measured at the shoot level, for drought adaptation (see Vadez, 2014).

Historical breeding programs have been mainly focused on visible and easily quantifiable traits such as grain production, shoot biomass, or resistance to diseases and pests, all accessible traits from aboveground parts of the plant (Paez-Garcia et al., 2015). Breeding programs emerging from the Green Revolution have not directly focused on the root system. As root architecture was shown to be positively correlated with plant productivity (Lynch, 1995; Kell, 2011; Hufnagel et al., 2014), there is an increasing interest in developing plant breeding programs directed at “improving” RSA and developing new cultivars with higher soil resources exploitation efficiency or better tolerance to environmental stress such as drought (Wasson et al., 2012). For example in sorghum, QTLs for nodal root angle have been identified and shown to co-locate with QTL for traits related to drought adaptation (Singh et al., 2010, 2012; Mace et al., 2012). However, carrying out root traits-based breeding calls for identifying root-specific phenes related to the optimization of soil exploration and water and nutrient uptake in various environments (Lynch et al., 2014). A prerequisite for this is to be able to phenotype and to select desirable root parameters (Paez-Garcia et al., 2015). However, root systems have long been neglected in breeding programs specifically because they are hard to phenotype. The rising interest in root breeding has thus sparked the development of a wide spectrum of root phenotyping techniques covering a large panel of growth conditions (Paez-Garcia et al., 2015). Those techniques can be used to characterize and quantify root growth and development parameters necessary for breeding programs. An example of such technique in the scope of drought research are lysimetric systems, where plants are grown in large tubes offering space and soil volume similar to field conditions. There, roots are not extracted destructively but plant water extracted by the roots—i.e., their functionality with regards to water—can be dynamically monitored throughout the crop cycle (Vadez et al., 2014).

In addition to the emergence of numerous varied phenotyping systems for roots, traditional breeding approaches can now also benefit from the mechanistic understanding coming from the field of functional-structural plant modeling (Xu et al., 2011). Mechanistic modeling approaches offer the possibility to integrate knowledge of plant development and physiology and to assess it against varied environment, leading to more reliable breeding (Lynch, 2013, 2015; Lynch et al., 2014). Functional-structural plant model (FSPM) approaches focus on the modeling of development, growth and function of all parts of the plant (cells, tissues, organs…) at different level of details in their spatio-temporal context. FSPMs are models that rely on an explicit and accurate description of the considered plant structure, and their efficiency is consequently tightly linked to the progress of phenotyping techniques. FSPM link the structure of the plant to its physiological processes, which are themselves driven by environmental factors (Godin and Sinoquet, 2005; Vos et al., 2007, 2010). The development of FSPMs is interdisciplinary by nature and uses various concepts, tools and software originating from a wide range of disciplines (DeJong et al., 2011). It can involve scientists with backgrounds ranging from plant physiology, plant development, soil science, mathematics, computer science, cellular biology, physics, to ecology and agronomy. For instance, to encode multiscale plant architecture, Godin and Caraglio (1998) used nested graphs which originated from mathematics and have been extensively used in others fields such as economy, networks and telecommunications, genetics and physics. Beyond encoding the plant structure, this multiscale formalism can also be used to simulate the development of plant architecture (Boudon et al., 2012; Ong et al., 2014).

Using FSPM to model the behavior of a crop root system can help understanding the extent of the impact of RSA on a given physiological process (DeJong et al., 2011). *In silico* approaches offer the advantage of fully mastering the studied system and allow to accurately assess the influence of each parameter on its functioning through sensitivity analysis (Han et al., 2012). FSPM have notably been used to simulate and study the development of plants in the context of water acquisition and transportation (Doussan, 1998; Roose and Fowler, 2004a; Doussan et al., 2006; Javaux et al., 2008; Couvreur et al., 2012; Lynch et al., 2014). In a breeding context, FSPMs can be very useful as they use a reverse-engineering approach to identify plant mechanisms likely to be beneficial under specific stress environment scenarios. In this review, some examples will be covered showing how crop simulation models can predict the effect of certain rooting traits on crop performances across time and geographical scale (e.g., Vadez et al., 2013; Kholová et al., 2014). FSPMs can also serve as a basis for the development of ideotypes by highlighting the parameters most likely to influence the adaptability to environmental constraints (Lynch, 2013; Lynch et al., 2014).

We focus here on the design of FSPMs that can be used in the broader context of research on root-related drought tolerance. First we will present the different phenotyping techniques existing for root architectural and physiological study and their limits, and will go over the root traits of interest for breeders. We will then present the integration of the generated data within architectural models, and how those data-driven architectural models can be coupled with functional hydraulic models useful for breeding studies. Finally we will discuss the assessment and validation of FSPMs hydraulic models through confrontation of simulations to experimentations.

## Root system phenotyping methods

Designing a functional-structural plant model (FSPM) presupposes to gather data related to plant structure and physiological processes that will serve as inputs to feed the model (DeJong et al., 2011). Plant phenotyping is the process of identifying and recording qualitative and quantitative traits that are depicting plant development and its functional aspects at different levels of organization (cell, tissue, organ, whole-plant scale) (Granier and Vile, 2014). Phenotyping strategies include skills and techniques that allow monitoring plant development and its response to different growth conditions in order to describe a full architectural and/or physiological outline in time and space. Many phenotyping techniques ranging from laboratory and greenhouse to field-based methods have been developed over the recent years (Paez-Garcia et al., 2015) and while most of them were applied to plant shoots (Berger et al., 2012; Araus and Cairns, 2014), a number of those allow for the characterization of root architecture.

The choice of a root phenotyping system depends on several factors, among others, plant species (annual vs. perennial), targeted traits of interest, studied developmental phase of the plant (early vs. terminal), necessity to gather 2D or 3D data, possibility to sacrifice the plant (destructive vs. non-destructive measurements), time scale of the growth kinetics (days vs. months) and costs (Paez-Garcia et al., 2015). The diversity of root phenotyping systems that have been developed over the years now allows researchers to choose the setup most adapted to their questions of interest (Kuijken et al., 2015) (Table 1).

**Table 1:** Overview of existing root phenotyping systems.

**Plant cultivation system**	**Growth media (localization)**	**Throughput**	**Destructive and dimensionality**	**Description**	**References**
1. X-Ray computed tomography	Soil (lab and greenhouse)	Very low (single plant at a time)	No/3D	This technique use X-ray to image root structure within a soil column. It generates stacks of projections which need to be combined and analyzed to reconstruct the 3D structure of the root system.	Mooney et al., 2011; Mairhofer et al., 2012; Mairhofer and Zappala, 2013; Koebernick et al., 2014
2. Shovelomics	Soil (field-based)	Low (Single to tens of plants in parallel, depending on available workforce)	Yes/3D	As the name imply, this method involves the manual and/or mechanical excavation of plants root systems from the soil. Roots can be measured *in situ* while being excavated, or phenotyped after washing and preparation.	Trachsel et al., 2011; Bucksch et al., 2014
3. Rhizotrons	Substrate (lab, field)	Low to medium (up to tens of plants in parallel)	No/2D	Rhizotrons are composed in principle of a succession of plates enclosing a thin layer of substrate. One at least of the external plates is transparent, and the rhizotron is built so that the root system grows in part or in total against this transparent plate, allowing for its imaging. In field conditions, the rhizotron can actually be a full trench along which the root system growth is observed.	Colin-Belgrand et al., 1989; Neufeld et al., 1989; Singh et al., 2010, 2012
4. Rhizolysimeters	Soil (field-based)	Low to medium (Tens to hundreds of plants in parallel)	No/3D	Rhizolysimeters are concrete, steel or PVC columns which are filled with soil and used to grow plants. The column can either be equipped with sensors or “windows” allowing for the observation and measurement of the plant as it grows by.	Eberbach and Hoffmann, 2013
5. Minirhizotron	Soil (field-based)	Low to medium (Tens to hundreds of plant in parallel)	No/3D	This particular system is based on transparent observation tubes which are permanently inserted in the soil. These tubes allow for the passage of a camera to image roots growing along the minirhizotron wall.	Iversen et al., 2012; Maeght et al., 2013
6. Growth and luminescence observatory (GLO-Roots)	Soil (lab)	Medium (tens of plants in parallel)	No/2D	Derived from the rhizotron principle, this system makes use of bioluminescent transgenic plants to image the growth of the root in soil.	Rellán-Álvarez et al., 2015
7. Rhizoscope	Liquid medium + solid support (glass beads) (lab)	High (hundreds of plants in parallel)	No/2D	This system is akin to a rhizotron. The main difference is that the growth substrate is replaced by transparent glass beads between which liquid medium is circulated. The glass beads can be removed to expose the root system for easy imaging and/or sampling.	Audebert et al., 2010
8. Clear pot method	Soil (greenhouse)	High (hundreds of plants in parallel)	No/3D	Again a variation on the rhizotron principle. Here plants are grown in transparent pots filled with soil or other potting medium. Seeds are planted close to the pot wall to enable high- throughput imaging of roots along the clear pot wall.	Richard and Hickey, 2015
9. Rhizoslides	Paper-based (lab, greenhouse)	High (hundreds of plants in parallel)	No/2D	This setup consists in growing the plants on germination paper supported by plexiglass plates and partially immerged in nutritive liquid medium, allowing for direct imaging of seedling growing on the paper.	Le Marié, 2014
10. Rhizoponics	Liquid medium (lab)	Very high (thousands of plants in parallel)	No/2D	Similar to rhizoscope systems in that it combines hydroponics and rhizotrons. The system is made of a nylon fabric supported by an aluminum frame. The set-up is immersed in a tank filled with liquid media.	Mathieu and Lobet, 2015
11. Root aeroponics	Air (lab)	Very high (thousands of plants in parallel)	No/3D	In this system plant are grown out of any kind of substrate and root are subjected to regular misting to provide water and nutrient. The root system is fully accessible at all time, albeit slumped due to growing without mechanical support.	de Dorlodot et al., 2005

*Adapted from Paez-Garcia et al. (2015)*.

One simple way that can help to categorize and choose among root phenotyping systems is to consider them from a throughput point of view, throughput being estimated both by the scaling of the experimental setup (how many experimental units can be deployed in parallel), and the time it takes to collect data per experimental unit. Lab and greenhouse-based phenotyping systems tend to allow for high-throughput phenotyping experiments (several hundred to several thousands of plants in parallel and/or quick data acquisition), allowing to test large number of seedlings in highly controlled and repeatable conditions (Table 1). These high-throughput methods are critical for QTL or GWAS studies aiming to link the plasticity of the RSA to genetic markers or specific genes or alleles that may be breeding target of interest. Medium throughput systems can typically deal with tens of plants at the same time and usually focus more on the spatial and temporal resolution of the data harvesting. Whether, they are lab or field based, these systems are often used to generate the architectural and physiological parameters used both for FSPM calibration and validation. On the lower throughput scale are methods requiring either costly technological tools (e.g., x-ray tomography) or significant data acquisition time (e.g., fine scale shovelomics). In addition to the low throughput, root x-ray tomography is still not perfectly mastered, being subjected to potential loss of information and added noise due to the low resolution of the generated images (Mooney et al., 2011) and the fact that automated 3D reconstruction of root system is carried out based on statistical modeling approaches (Mooney et al., 2011; Kuijken et al., 2015).

An important parameter to take into account when choosing a root phenotyping system is the balance between the need for controlled conditions and observation of the “real” development of the root. Lab and greenhouse based methods such as rhizotrons often constrain the root system growth into a 2D structure of limited size, which can rapidly impede root system growth. On the contrary, systems allowing for permanent accessibility of the root for observation and sampling (e.g., hydroponics and aeroponics) imply a lack of mechanical medium to support the RSA and to impact on its development, meaning that the pertinence of observed architectural phenotypes in those setups is debatable. While theoretically less structurally limiting, field-based methods need specific setup such as rainout shelters and irrigation systems to offer controlled conditions and to precisely take into account environments effects on root development, as well as strongly limit the extend of possible root system observation and measurement (Paez-Garcia et al., 2015). Intermediate strategies such as rhizolysimeters can offer rather unlimited growth under controlled (or at least monitored) conditions, but they require substantial structural investment to be practical.

Plant structure phenotyping procedures can typically be separated in three phases: firstly the acquisition of the architectural (and/or physiological) data within the phenotyping system of choice through imaging, secondly the analysis of the generated image to extract quantitative data regarding the characters of interest, and thirdly the subsequent analysis of this quantitative data to extract meaningful information such as mathematical laws describing a growth process. Regarding root architecture characterization, the first step is mainly limited by the difficulty of accessing to root systems either visually or physically, an issue for which several solutions have been devised (Table 1). The second step however is generally highly dependent on image analysis capacities and constitutes the main bottleneck of root phenotyping studies (Furbank and Tester, 2011).

From low to high throughput phenotyping systems, morphological and structural information is mainly generated as 2D images that need to be processed into quantitative data representing a 3D structure (Kuijken et al., 2015). And because the root system is a 3D structure (even in 2D rhizotron), any sufficiently old root system will exhibit overlapping of roots in 2D projection pictures. This greatly complicates the extraction of root structure from images and has lead to the development of a wide range of image analysis algorithms and software applications to help automatically extract root structure from noisy images. These image analysis software usually offer functions to quantify root features such as root number, length or angles which can be used to calibrate or validate root FSPM (Godin and Sinoquet, 2005; Vos et al., 2007, 2010; Lynch et al., 2014). Kuijken et al. (2015) recently reviewed all currently available image processing software applicable to root phenotyping. Their increasing number results in a large variety of software solutions for root systems analysis (Lobet et al., 2013). However, this diversity also led to the proliferation of independent computational methods and framework to represent and store root architectures. It is a hindrance that limits the possibility to exchange data between labs or to use different software on the same dataset. To resolve this issue, a common root architectural description was recently developed. Emerging from an international joined effort by several groups working in root phenotyping and modeling, the Root System Markup language (RSML) has been specified to ensure root architecture data transferability between software, thus promoting research exchanges within the scientific community and given rise to a standard format upon which to build central root model warehouses (Lobet et al., 2015).

## Root architecture phenotyping in a breeding context

The phenotyping methods described above are still not widely use in the context of breeding programs, in part because the link between measurable traits and their usefulness in the context of breeding is not always evident, and in part because of somewhat limited throughput of analysis compared to the genomics methods of analysis that can be used to support breeding programs such as GWAS for example (based on thousands to tens of thousands of plants) (Spindel et al., 2015; Biscarini et al., 2016; Gao et al., 2016; Iwata et al., 2016). Yet, breeding effort targeting several aspects of the RSA have been successfully undertaken in different crops (Table 2). For example Tuberosa et al. (2002a,b) identified QTLs for seminal root traits in a maize recombinant inbred line population and found a certain degree of co-location between QTLs for seminal root traits and QTLs for yield performance across different water regimes in the field. In chickpea a major QTL for root traits (depth, density) was identified (Varshney et al., 2014), from phenotypic data generated in a PVC tube system where plants were grown and root extracted and scanned at 35 days after sowing (see Kashiwagi et al., 2005 for a method). In sorghum, genotypic variation for nodal root angle was identified (Singh et al., 2010, 2012) and these traits are seen as a potential target for breeding program for either deep rooting (narrow angle), or rooting in the scope of skipped-row planting that requires shallow root angle. Subsequently, a phenotyping platform was developed at a scale that allowed phenotyping of a mapping population and QTLs for nodal root angle have been identified and shown to co-locate with QTL for traits related to drought adaptation. These three examples, taken from a wider variety of uses of root traits in breeding (Table 2) illustrate how simplified techniques (i.e., a hydroponic system, or root angle measurements between two thin plates) can be sufficient to pinpoint genotypic variation in traits that are strongly related to field-based performance.

**Table 2:** Structural and functional root traits identified as potentially relevant for drought-resistance breeding.

**Traits**	**Species**	**QTLs**	**Sources**
Root length	Rice, Wheat, Maize	Yes	Price et al., 2002; Tuberosa et al., 2002a,b; MacMillan et al., 2006; Courtois et al., 2009
Root biomass	Rice	Yes	Courtois et al., 2003
Root thickness	Rice, Maize	Yes	Zheng et al., 2000; Tuberosa et al., 2002a,b
Total root biomass	Wheat, Maize	Yes	Tuberosa et al., 2002a,b; Sharma et al., 2011
Root length density (RLD)	Chickpea	No	Kashiwagi et al., 2005
Seminal root angle	Wheat	Yes	Christopher et al., 2013
Number of seminal roots	Wheat	Yes	Christopher et al., 2013
Crown root angle	Maize, Sorghum	Yes	Giuliani et al., 2005; Singh et al., 2010, 2012
Rooting depth	Wheat, Chickpea	No	Sayar et al., 2007; Varshney et al., 2014
Crown root diameter	Maize	Yes	Giuliani et al., 2005
Xylem vessel size and number	Rice, Wheat	Yes	Richards and Passioura, 1989; Uga et al., 2008
Root cortical aerenchyma	Maize	Yes	Mano and Omori, 2009

## Root system architectural modeling

The ability of roots to ensure the hydro-mineral nutrition of the plant is dependent on RSA (Lynch, 1995, 2007; Comas et al., 2013; Lynch et al., 2014), but also on the root hydraulic characteristics. Root systems appear highly plastic, and their structure is the result of complex interactions between genetic and environmental regulations. Those interactions generate dynamic feedback loops in which the heterogeneousness of the soil environment modifies the plant growth, which in turn modify the soil by harvesting water and nutrient from it, and so forth. One way to investigate and solve such complex feedback system is to use models.

Being hidden underground, root systems are particularly challenging to model. Nevertheless, a lot of architectural root models have been developed over the last 40 years. In all cases, the very first step of the modeling process consists in choosing an adequate representation (i.e., formal encoding) for the root structure.

Due to the inherent difficulty to assess the precise root spatial distribution in soil, the first root system architectural model where actually continuous models based on estimates of root density distribution within the soil through time and depth (Dupuy et al., 2010). An early example of such models used diffusion equation to model the dispersion of root within the soil (Page and Gerwitz, 1974). However, density-distribution models relied on synthetic parameters such as a single root density descriptor and were based on the hypothesis that roots distribute regularly throughout the soil. Such an assumption is not verified in field conditions where a discontinuous distribution of roots is observed, presumably due to heterogeneous distribution of environmental effects. As such, simple continuous models cannot easily take into account effects such as root clustering which is instrumental for resource uptake (Dupuy et al., 2010). As a consequence, rather than focusing on the precise developmental regulation of RSA, these types of continuous architectural models are better suited to give synthetic descriptions of RSA in global environments. As they can be used to infer missing or imprecise architectural information, continuous models are best used to investigate RSA when root systems are partially or totally inaccessible, for instance when studying mature trees or field grown plants. In order to be able to investigate RSA in heterogeneous soil conditions in the field, continuous model can be further coupled with statistical approaches allowing for the description of root density statistical maps through the soil (Chopart and Siband, 1999). The main limitation of those coupled models is their reliance on calibration data that need to be generated from tedious *in situ* excavation and manual measurement of different parts of the root system in soil (Chopart and Siband, 1999).

RSA emerge from the interaction between root developmental processes and their environment. As they do not consider individual roots, continuous models cannot easily account for the feedback existing between root and soil. Therefore, new approaches were needed to understand how soil is explored by the plant at the individual root axis level (Pierret et al., 2007). This consideration gave rise to the development of more complex root models. Those models are based on the explicit description of root development, growth and branching processes resulting in 1D, 2D, or 3D models (Dunbabin et al., 2013). Such discrete and explicit models consider root architecture through its complete discrete topology and geometry and can be based on several distinct mathematical formalisms (Godin, 2000; Balduzzi et al., 2017). Two of the most popular formalisms used to represent discrete plant architecture in general are multi-scale tree graphs (MTGs) (Godin and Caraglio, 1998; Godin et al., 1999, 2005) and L-systems (Prusinkiewicz and Lindenmayer, 1990).

MTGs were developed based on the concept of plant modularity and aim to describe individual parts of the plants as tree graphs, themselves included in an arborescent structure (Godin et al., 1997). MTGs allow topological and geometrical encoding of any kind of plant and were used as a standard to describe plant development and architecture of a broad range of species (Godin and Caraglio, 1998; Danjon et al., 1999; Godin, 2000; Guédon et al., 2001; Danjon and Reubens, 2008; Fournier et al., 2010; Garin et al., 2014; Griffon and de Coligny, 2014). The MTG formalism has notably been used as the principal data structure for the OpenAlea platform (http://openalea.gforge.inria.fr), a software environment dedicated for plant modeling which integrates algorithms designed for creating, parsing, modifying and extending MTGs (Pradal et al., 2008, 2015), as well as algorithm to convert MTGs to the recently defined RSML formalism and conversely (Lobet et al., 2015).

While MTGs can be extended to provide dynamical consideration of plant architecture, they are inherently static structural descriptions. Another way to encode plant architecture is to see it as the result of iterative developmental processes and try to express it using a procedural formalism. This is the view chosen in the L-systems formalism (Lindenmayer, 1968). This formalism uses a symbolic language to provide a description of the plant as a bracketed string of characters. Each character stands for a given plant developmental module (meristems, organs, metamers, segments, axes, etc.). Developmental rules are specified as rewriting rules for each possible type of characters, indicating whether it stays the same or is replaced by another character or group of characters at each iteration. The repetitive and recurrent nature of plant structure thus allow to capture and to recreate plant developing architecture through time by discretizing the plant as a set of characters and specifying a reduced set of rewriting rules (Prusinkiewicz, 2004).

Since their first formalization, L-systems have been implemented and extended through different modeling languages and systems, notably cpfg (Prusinkiewicz and Lindenmayer, 1990; Prusinkiewicz and Karwowski, 1999), lpfg (Karwowski and Prusinkiewicz, 2003), XL (Kniemeyer and Kurth, 2008) and more recently L-Py (Boudon et al., 2012). This latest installment of L-system formalism implementation was designed to allow mutual conversion between L-strings and MTGs. This offers the possibility to use the large set of available built-in components, tools and algorithms already designed for MTGs in conjunction with L-systems (Boudon et al., 2012). While both structural and procedural formalisms were initially designed with plant aerial part structure in mind, both have been used with success to generate discrete explicit root system models. For instance the mature RSA of *Pinus pinaster* was reconstructed from 3D-digitizing data using a MTG approach (Danjon et al., 2005). In the same species, MTG-type simulated root systems were used to investigate plant anchorage and its response to architectural modification (root wounding, absence of tap root, pruned root systems, etc.) (Khuder et al., 2007; Danjon and Reubens, 2008). Another example is Root Box (Leitner et al., 2010b) that represents root growth and architecture using L-systems. It is encoded in Matlab and was applied to the study of maize root system. This model uses a modular approach to integrate the interplay between root and soil and can be used to compute complex root system properties such as root length density distribution for different soil models (Leitner et al., 2010a). The model code is publicly available (http://www.boku.ac.at/marhizo/simulations.html) and has already been coupled with different soil models to simulate the influence of chemotropism on root growth (Schnepf et al., 2011), the effect of root exudation on phosphate acquisition (Schnepf et al., 2012), or the impact of root architecture on water acquisition under different hydrological conditions (Tron et al., 2015).

Beyond MTGs and L-systems, another formalism is the fractal approach, which uses mathematical concepts initially developed for the study of geometric patterns in nature and, in particular, to characterize self-similar patterns. This specific formalism has notably been used to develop a static 3D architectural model of *Gliricidia sepium* root system (Ozier-Lafontaine et al., 1999). This model was able to efficiently predict root branching patterns and some root traits at plant level such as root dry matter, total root length and root system diameter (van Noordwijk and Mulia, 2002; Doussan et al., 2003).

From a breeding point of view, all those encoding formalisms give convenient access to root structure descriptors and allow for easy quantification of plant root system shape. However, by themselves they are not enough to understand how the root structure emerges and thus need to be coupled with mechanistic developmental rules. In addition, once proper formalisms are integrated in root models, and their functions validated, the conditions in which the root phenotypes that emerge from these models have a demonstrated effect on crop productivity will have to be validated. For instance in maize, it was shown that reduced lateral root branching was beneficial for crop adaptation to water stress because of a reduced carbon cost of that root type (Zhan et al., 2015). In such case, a clear link could be conceived between some well-defined encoding formalism and its expression in the form of a phenotype of importance for a breeding perspective.

**Figure 1:**
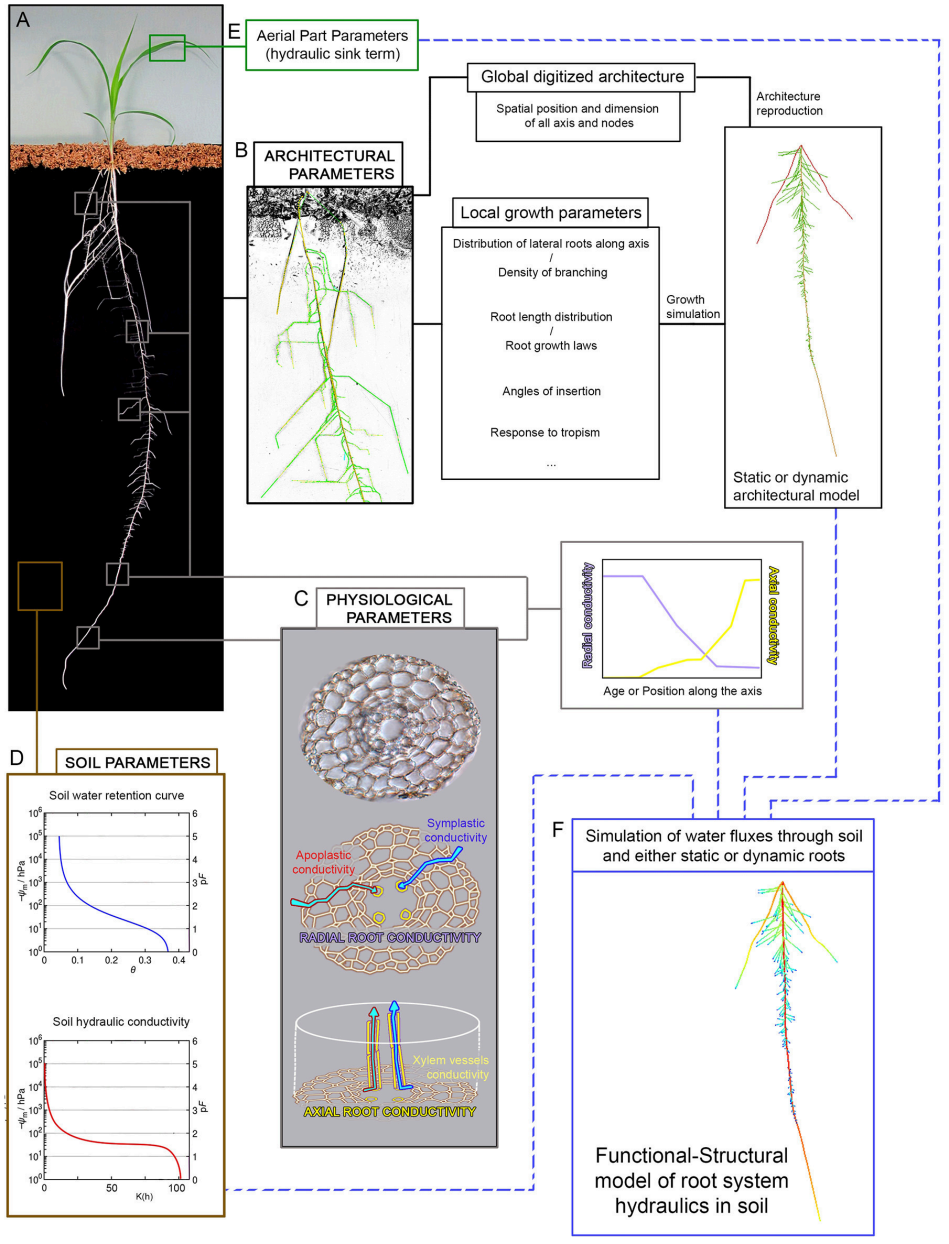
Parameters acquisition for root hydraulics models. The generation of root hydraulics models requires several set of parameters which are often model-specific. In the ideal case, those parameters are generated from phenotyping experiments. **(A)** The type of phenotyping setup available (see Table 1) will condition the type and amount of data usable for parameter definition, but in all cases they are at least able to provide root architecture data. Generally speaking, complete root hydraulics models including a soil compartment, such as R-SWMS for example, need four types of parameters. Architectural parameters acquired through image analysis of the root structure **(B)** can be used in two distinct ways: either the whole root architecture is digitized and the root structure is reproduced computationally, or the root architecture is used to determine local growth parameters which can in turn be used to create representative root architecture through growth simulation. Physiological parameters **(C)** relating to water transport are principally acquired through additional histological and physiological measurement. Radial root conductivity is a function of apoplastic and symplastic water transport and is hard to evaluate, often needing to be estimated through proxy such as pressure probe measurement in outer cells layers. Axial root conductivity is dependent on xylem vessel size and shape and can be partially extrapoled from cell measurement and application of Hagen-Poiseuille's law. Those two parameters need to be evaluated along the root axis and/or for different root ages to generate profiles of conductivity. Soil parameters needed **(D)** are soil water retention and soil hydraulic conductivity profiles, as well as an eventual description of the soil structure. Finally, depending on the model, aerial part parameters **(E)** can be more or less explicit and are used to express a hydraulic sink term driving water absorption by the root. Taken altogether, these four sets of parameters can be used to simulate the dynamic of water fluxes through the soil and roots and to study the patterns of water distribution under a given environment **(F)**.

Many 3D mechanistic dynamic root architectural simulation models have been developed since the 80s to investigate root growth and function (Dunbabin et al., 2013). They usually rely on the description of different mechanistic developmental rules depending on root branching order and/or root type. Those rules need to be calibrated against data obtained through large-scale phenotyping or specific experimental measurements. Different root types can be characterized through developmental descriptors or criteria such as growth rate, branching variability, branching density, tropism efficiency, radial growth, etc. Each criterion is considered a distinct parameter for the generation of the 2D or 3D structural models (Figure 1, Pages, 2002). Development of the whole root system is simulated in discrete time points on the basis of the specified morphogenetic rules (Doussan et al., 2003; Prusinkiewicz, 2004). These rules govern initiation (branching), emergence and growth of new axes but can also integrate rules for root senescence and / or rule describing the influence of various tropisms on root development (thigmotropism, hygrotropism, chemotropism, gravitropism, …). One of the first 3D mechanistic root model was developed by Deans and Ford (1983). This model was able to simulate a 16-year-old excavated root system of *Sitka pruce* and allowed investigation of wind impact on the tree development and stability (Deans and Ford, 1983). It inspired the development of subsequent 3D mechanistic root models of others species using the same method to describe the elementary growth and branching processes of root systems. An example of such later model is ROOTMAP (Diggle, 1988a,b) that simulated root growth and architecture of fibrous root systems. It considered mechanistic parameters for growth (e.g., root-elongation rate) and branching (branching angle, branching density, time of branching delay, branching order, etc.). This model was used to simulate a broad array of lupin genotypes with a high accuracy using data acquired from semi-hydroponic phenotyping system (Chen et al., 2011). It was since then extended to integrate a 3D soil model, thus representing root system plasticity in a mixed soil environment and allowing to model nutrient uptake dynamics from that environment (Dunbabin et al., 2002). Pagès et al. (1989) used the same approach as Diggle (1988a,b) to produce a 3D RSA model of maize using empirical observations to define morphogenetic rules and different growth processes depending on root branching order and inter-node root origin (Pagès et al., 1989). Based on the concepts developed by previous models (Diggle, 1988a,b; Pagès et al., 1989), the SimRoot model was designed with better focus on visualization, taking into account the spatial heterogeneity of root growth processes through a kinematic description of variation of growth features along root axes (Lynch et al., 1997). It has been calibrated using empirical datasets acquired from different growth environments and was used to predict precisely the growth of maize and bean root systems (Ge et al., 2000; Postma and Lynch, 2010). SimRoot has also been extended to integrate interactions between root systems, phosphorus uptake efficiency (Ma et al., 2001), carbon allocation (Nielsen and Lynch, 1994; Walk et al., 2006; Postma and Lynch, 2011) and shoot/root exchanges by coupling with the LINTUL model (Postma and Lynch, 2011; Dunbabin et al., 2013). Another generic mechanistic root model is RootTyp (Pagès et al., 2004). Contrary to previous models that differentiated root behavior depending on their branching order, RootTyp relies on the explicit determination of different root types (with different growth properties such as branching density or elongation rate) independently of their branching order (Pagès et al., 1989). RootTyp has been used to represent a large variety of plant root systems and was parameterized using various architectural datasets (Collet et al., 2006; Garré et al., 2012). It integrates stochasticity through inclusion of randomness in some geometrical or topological parameters (e.g., root trajectories). As of particular interest in the context of study of root-driven drought tolerance, it is to be noted that RootTyp was also extended to provide dynamic description of water supply within the soil environment (Doussan et al., 2006; Draye and Pagès, 2006).

One common denominator for all those mechanistic models to be useful as predictive tools is their dependence on the previously described phenotyping methods (Table 1) to provide the architectural data necessary both for model rule calibration and for validation of model predictions (Figure 1). Architectural data can be generated using any of the described phenotyping platforms, as each of those allow for the possibility to capture the structure of the root system in some way. Depending on the considered model and on the nature of the image analysis procedure following root system imaging, parameter calibration can be done from global descriptors (for example statistical distribution of root densities in the soil, or on the contrary the entire and precise description of the root structure) or from quantitative measurement of local specific architectural traits or growth laws (root densities, root length distribution or root growth speed, root angle of insertion, etc). In ideal cases, at least two independent datasets will be used for parameter definition, one to calibrate the model and one to validate its predictions.

Following parameter definition, the models are expected to provide digital root architectures based on the input parameters, either reproduced from structural data or digitally grown from local growth laws. These simulated architecture need then to be validated against the additional datasets. Depending on the model and on the nature of these datasets, this validation can be done either by direct structural comparison of architecture, or through the use of indirect descriptors (for example, amount of root biomass by soil horizons, distribution of root length between the different root orders, etc). In some specific circumstances, only a single dataset may be available both for calibration and validation. In this case, it is still possible to proceed by calibrating the model using only part of the available data, validating it against the rest of the data, and repeating this procedure for all possible combination of sub-dataset.

## Conception of functional-structural models of root hydraulics

We have gone over the different solutions available to gather data on root structure through phenotyping, how this data can be encoded using various mathematical formalisms and then used to provide calibration and rules for various mechanistic models of root development. Those mechanistic developmental models can then be further coupled with functional processes in order to provide an integrated view that could ultimately support model-assisted breeding programs (Figure 1).

One such functional process is water uptake and transport by plant roots. Drought is one of the mains limiting factors for plant productivity (Lobell and Gourdji, 2012), therefore using models to understand where, when and how water is absorbed and transported from the soil by the plant roots could help improving water use both by optimizing plant RSA through ideotype-assisted breeding and through model-directed changes in agricultural management practices (Lynch, 2007; Blum, 2009; Palta et al., 2011; Lynch et al., 2014). It would be also important to couple these modeling approaches with experimental setup that allow a very precise evaluation of water extraction. For instance, the use of a lysimeter system for close monitoring of plant water use (Vadez et al., 2014) has shown genotypic variation in sorghum germplasm for the capacity to extract water from the soil profile (Vadez et al., 2011). It would be interesting to investigate which rooting trait or traits can explain the contrasting water extraction characteristics and whether these can be exploited in further breeding programs. Such traits may be macroscopic and related to the repartition of different root types in the soil, or microscopic and linked to cellular processes or structure (e.g., root hair or xylem cells size, changes in tissue conductivity through differential aquaporin expression, etc…).

The dynamics of water uptake from the soil is a very complex issue which depends both on the properties of a dynamic biological system (the root) and of a physical heterogeneous system (the soil). Studies of water dynamics in soil were initiated in the 1960s with the innovative work of Gardner (1960) which was more focused on soil than plant properties but nevertheless served as the basis for subsequent works to better understand root-soil exchange processes. These later works introduced roots in the form of sink terms in soil water distribution models to represent soil water uptake by roots (Feddes, 1974; Molz, 1981; Homaee et al., 2002; Dardanelli et al., 2004). Those models mainly use a continuous root system representation, describing it through root length density descriptors. As the feedback between root system growth and its environment has been proven to play a major role in the dynamics of soil resources uptake (Doussan et al., 2003; Lynch et al., 2014), FSPMs had to be able to deal with developmental feedback.

Specific root-soil FSPMs have been developed to investigate the interaction between soil water hydrodynamics and root system development. Clausnitzer and Hopmans (1994) proposed a detailed FSPM-type root hydraulic model that coupled a 3D root architectural model with a 3D transient water flux model. They modeled the interaction between root growth and soil water distribution to simulate water uptake in crops. In their model, processes governing root development are expressed as a function of local soil conditions, and the water sink root uptake term is expressed as a function of transpiration and root length. The soil itself is discretized and a finite-element grid is used as the basis for soil properties computation. Benjamin et al. (1996) combined a 2D root model of corn root system with a 2D water model to simulate the effects of root patterns on water uptake. Somma et al. (1998) extended the model of Clausnitzer and Hopmans to express water uptake activity of roots as a function of root age and to additionally simulate solute transport and nutrient uptake.

Beyond those models based essentially on soil water fluxes, the dynamic of water fluxes in the plant tissues was also the focus of early modeling studies where plants were considered using an electrical analogy (van den Honert, 1948). In this first modeling effort and subsequent works, plant tissues are represented using a network of hydraulic resistances behaving as electrical resistances, and water transport is governed by pure physical consideration (van den Honert, 1948; Zimmermann, 1978). The parameterization of those models however required to be able to determine the value of the hydraulic resistance of plant tissues. This lead to the extensive development of measurement methods allowing for the estimation of physiological parameters of water transports in tissues, attested by the extensive work of groups such as those of Steudle (Hüsken et al., 1978; Steudle, 1993, 2000) and Sperry (Sperry et al., 2002; Sperry, 2011; Sperry and Love, 2015). Interestingly, these measurement methods essentially allow for the determination of water conductance rather than resistance. As those two physical values are inversely linked, models of water transport in plant tissues based on electrical analogy can use either value for their parameterization, provided that the equations are accordingly tweaked. Those models essentially need two types of conductances (or resistances) for their parameterization: namely axial and radial conductances (resistances) (Figure 1). Axial conductance is directly linked to the structure of the vascular tissue. Under the assumption that the vascular strands are lengthy regular cylinders, it can be directly computed from application of generic Hagen-Poiseuille law. This predicted axial conductivity values may need to be adjusted depending on the proportion of embolized vessels existing in the considered species. Computation of radial resistance on the contrary will depends the level of details of the considered model, as it is the results of the combination of conductances of the cells comprised in the concentric tissue layers of the plant axis.

In 1998, Doussan et al. proposed a novel approach integrating in a single framework knowledge about water flux in the soil, plant water uptake, plant vascular structure, global root tissue conductivities and RSA. In this approach, the architectural model of Pagès et al. (1989) was combined with the biophysical description of water fluxes in root tissues considered as a network of radial and axial water conductances. Calibrated using both measured and estimated conductances from tree root phenotyping, this model can simulate root water fluxes through computation of water potentials along the conductance network. Lately, Couvreur et al. (2012) extended this approach by computing analytical solutions to water flow equations for complex hydraulic architecture in simulations of water fluxes distribution under drought. Chopard (2004) simulated water transfer in soil and root systems using a 3D root architectural model based on MTG formalism and integrating water transport processes within differentiated root types. Integrated models can also result from the conjunction of several pre-existing independent models. For instance, Javaux et al. (2008) developed the R-SWMS model from the conjunction of the models of Doussan et al. (1998, 2006) and Somma et al. (1998), coupling a mechanistic root development model with deep integrated knowledge of soil hydrology processes. R-SMWS can be used to simulate various water distribution and uptake rules under a wide variety of environmental conditions (Draye et al., 2010; Couvreur et al., 2012). Beyond being used to estimate water absorption by the roots, those models can also be extended to study nutrient uptake, such as was done by Roose and Fowler (2004a,b) in the case of phosphate uptake. Latest modeling development also focuses on the crucial problematic of model scaling and extrapolation (Meunier et al., 2017). The majority of plant root and soil hydraulics models are designed and parameterized from experimental data generated in lab or greenhouse, while they should ideally be intended to give prediction regarding the behavior of field-grown plants. Meunier et al. (2017) recently proposed a numerical solution to extrapolate global root behavior from sets of local variables that can be easily measured in lab or greenhouse. However, this solution can only be applied to root systems presenting a highly regular structure, and the problem of model field-projection for irregular, life-like root systems still need to be addressed.

## Validation of root hydraulics functional-structural models

Root hydraulic FSPMs can be used to predict the dynamics of water distribution in the plant-soil continuum (Roose and Fowler, 2004a,b; Doussan et al., 2006; Javaux et al., 2008; Moradi et al., 2011; Couvreur et al., 2012). The quality of those predictions is dependent on the correct calibration of architectural and functional characteristics of roots and hydraulic properties of soil from phenotyping and physical measurement techniques. It is also known that the genetic of the plant can have profound role on the hydraulics of the root system. For example, Ehlert et al. (2009) showed different hydraulic conductivities in maize genotypes treated with a range of aquaporin inhibitors. Moreover, for the predictions to be of use in model-assisted breeding, they must themselves be validated against observable environmental and physiological parameters.

Validation methods of water fluxes prediction use various non-destructive and non-invasive imaging techniques to allow for the real-time observations of water content in root-soil systems (Doussan et al., 2006; Garrigues et al., 2006; Perret et al., 2007; Pohlmeier et al., 2008; Carminati et al., 2010; Moradi et al., 2011). The predictions of the Doussan et al. (2006) root hydraulic model were confronted to an experimental system aimed at monitoring dynamic water depletion around roots in soil (Garrigues et al., 2006). The principle of this system is to measure changes in light transmission value between different water saturation level of the soil matrix and use those to compute the uptake of water by the root system throughout the soil. This experimental setup is a derivative of rhizotron systems (Table 1) and can theoretically be applied to any kind of root/soil system, scaling up to fully grown crops such as mature maize root system. This setup showed that the prediction of the root hydraulic model were qualitatively and quantitatively representative of the water dynamics observed within the root-soil system, with greater water depletion occurring close to root base. Regarding phenotyping systems where the root architecture is not directly apparent but rather embedded in the soil, alternative techniques need to be used to quantify water movement through soil and roots, such as magnetic resonance imaging (MRI). Pohlmeier et al. (2008) used MRI to monitor changes in water uptake dynamics in soil. Both soil water content and root architecture can be imaged using this technique, and imaging results revealed that water uptake is greater in zones were the root densities are the highest. This technique is however still limited to imaging in-lab experimental setup of small dimension and is not yet directly applicable in field or for rhizolysimeters. The creation of portable MRI apparatus is one of the challenges that need to be addressed in order to be able to validate hydraulic model prediction in large scale and *in situ* phenotyping platforms. Alternatively to MRI, Carminati et al. (2010) used neutron radiography to image 2D water content distributions in soil under drought conditions and following rewetting in order to investigate the role of rhizosphere in water uptake and drought tolerance. While chemical and physical characteristics of the rhizosphere have been proven to be different from that of the bulk soil (Strayer et al., 2003; Gregory, 2006; Hinsinger et al., 2009), the effects of these specific properties on water uptake are usually neglected in root hydraulics models. 2D water distribution patterns observed using neutron radiography showed that the water content of the rhizosphere is higher than that of the bulk soil during drying and reversely during rewetting. These observations were used to determine the respective water retention curves of rhizosphere and bulk soil. Given these parameters, a simulation of water flux for a single root according to the model of Gardner (1960) suggested that the rhizosphere actually acts as a buffer to soften the impact of drought and provide smooth water availability in time of water stress. Moradi et al. (2011) further advanced this line of inquiry, using neutron tomography to quantify and visualize water content dynamics in 3D with high spatial resolution in the rhizosphere of three different plant species. They observed increased water content in soil next to roots (rhizosphere) and the observations were consistent in the three species (chickpea, white lupin and maize), confirming the conclusions of Carminati et al. (2010). The measured experimental water retention profiles were used in a simplified 3D analytical model which again confirmed the conclusion of the previous 2D model, highlighting the importance of the rhizosphere in water uptake processes and its potential interest as a target for drought-tolerance breeding programs. In another set of studies, Zarebanadkouki et al. (2012, 2013, 2014) used neutron radiography coupled with injection of deuterated water D_2_O to actually trace water fluxes within roots. D_2_O was injected into roots and its transport dynamics were tracked closely using time-series neutron radiography. To quantify the local transport of D_2_O through convective fluxes, a diffusive-convective model was developed, taking into account the different water pathway available in plant tissues (apoplastic, cell-to-cell). Model predictions on D_2_O fluxes were in harmony with experimental measures of axial flow of D_2_O inside the roots of 24-days old lupin plants.

In all those instances, the prediction of the different root hydraulics FSPMs could be validated against experimental data in the lab, further confirming the interest of such models to investigate the behavior of plant regarding water acquisition. However, those various validation methods all suffer from similar limitation in that they cannot easily be transposed to field-based measurement and thus are yet almost exclusively limited to the validation of lab-based and greenhouse-based predictions. This point, among others, constitutes one of the challenges that need to be addressed in order to promote the use of FSPM model in future breeding programs. In particular, a link needs to be made between the root phenotype that these models are able to predict, and root or plant phenotypes that would have a demonstrated effect on crop performance in the field conditions. Another limit of the current validation methods is the lack of way to generate anisotropic hydraulic environments in the existing phenotyping platforms. In order to precisely predict the impact of water availability in the environment on the root architecture, the models need to integrate rules expressing retroaction existing between root growth and water acquisition. These rules in turns need to be parameterized and validated against experimental data. Current phenotyping platform do not allow for easy control of the local hydraulic potential of the root environment: field based assays are limited to controlling global irrigation; rhizotrons grown plants are usually irrigated either from the top down or from the bottom up, resulting on a gravity induced water gradient in the soil; liquid media-based setup are by definition saturated in water and aeroponics systems are isotropic in term of water availability. They are currently several ways to improve on this situation and we will only list a few possibilities for some of those phenotyping systems, whether or not these are currently being pursued by different groups: rhizotrons can be improved by predefined subdivision of the soil into compartments with different hydraulics properties; another possibility would be to devise a setup to provide water at different points of the rhizotron, perhaps through distribution of capillary dripping through the plates of the setup—this last possibility could also be adapted to rhizolysimeters setups through differential water feed along the column; liquid based setup can be improved using water-retentive beads instead of glass beads as a mechanical support, modifying the proportion and/or distribution of those beads to affect the pattern of hydraulic potential around the root system. These new systems would allow the modeler to have access to data regarding the response of the root to changes in its environment and could also be used to facilitate testing for hypothetical drought scenario by selectively depriving parts of the root system from water. This would in turn help the models predict and validate the optimal root architecture for a given water-constrained environment.

## Linking models prediction to breeding

While high-tech phenotyping methods such as X-Ray tomography and models of functional root architecture can appear to be too theoretical to be of use in breeding studies at a first glance, there is actually a growing body of literature regarding models based on root phenotyping successfully predicting performance improvement associated with the selection of certain root traits. For example, a study in sorghum showed that the advantage of lines introgressed with staygreen QTL came from the capacity to restrict transpiration under high evaporative demand. The virtual crop model predicted a clear advantage from this trait in terms of grain yield. It was then found that this trait, measured at the shoot level, could be related to differences in the root hydraulic conductance and indeed contrasting lines were identified, having different root hydraulic characteristics (Kholová et al., 2014). Another example in maize used modeling to predict the maize yield changes in corn over the last century, and showed that changes in root architecture were the most likely reason for the increases in yield (Hammer et al., 2009). A modeling study in chickpea showed that increasing the speed of root growth, which related to rooting depth and rooting density, was likely to lead to a faster depletion of the soil water, bringing about a yield penalty (Vadez et al., 2012). On the contrary, increasing the depth of water extraction was the mean by which yield could be increased. While the former traits were related to root expansion and branching, the latter deals very likely with a different root architecture with more profuse rooting / branching at depth. Similar work was done in wheat, showing again the value of more profuse density at depth (Manschadi et al., 2006).

## Upcoming challenges of FSPM approaches

In the wake of global climatic changes and of increasing concerns regarding the limits of the agricultural methods inherited from the Green Revolution, a rising opinion is that a Second Green Revolution will actually come both from the consideration of plant roots and from the use of models and systems biology to promote more mechanistically-driven breeding and more rational agricultural practices (Lynch, 2007; Lynch et al., 2014).

We have seen here how FSPMs built upon the advances of phenotyping and modeling techniques can provide insight on the mechanisms of root development and water acquisition. Those predictive models have three-fold interests. First, they allow for quantification of the respective contribution of each parameter of the root system to water acquisition through sensitivity analysis approaches, and thus help focusing breeding efforts on the most important phenes. Second, they can be used to search through the plant structure-function-space for integrated root ideotypes best adapted to various environmental scenarios. Those ideotypes can then be used as target and guideline for subsequent breeding projects (Lynch, 2013). Third, they offer the opportunity to rapidly and cheaply assess the effect of alternative agricultural strategies *in silico* before deploying them to field assays. For instance, root hydraulics FSPMs can be used to test various timing and magnitude of irrigation strategies and to optimize these in regards to the dynamics of the plant water acquisition capacities. Yet, despite all those advantages, root hydraulics FSPMs are still faced with a certain number of challenges that need to be addressed.

We have already mentioned some of those challenges. For instance, automated image analysis is the current bottleneck of most root system phenotyping approaches (Furbank and Tester, 2011; Roose et al., 2016). This issue could be solved in two ways, either by diminishing the amount of noise in the generated data or by improving the analysis of noisy data. Diminishing the noise-to-signal ratio can be done by improving upon phenotyping systems to allow better visualization and capture of the root structure, using for instance technological advances such as plant MRI (Stingaciu et al., 2013; Metzner et al., 2015), neutron radiography (Leitner et al., 2014a) or simultaneous imaging of the root system at different angles to overcome the root overlapping issue. By contrast, the improvement of noisy data analysis will rely mainly on computer vision advances and on the development of novel signal-detection algorithms. Moreover, even with fully automated approaches, the plant community will face new challenges such as the reproducibility of computational experiments (Cohen-Boulakia et al., 2017) and the management of very large amount of data (Pradal et al., 2017). The development of new computational methods in Phenotyping (e.g., scientific workflows) and the availability of very large distributed infrastructure (i.e., cloud, grid) will be needed to tackle the new challenges that appear with the need to process very large amount of data in a n automated and reproducible way (Bucksch et al., 2017).

Another challenge is the difficulty to acquire data regarding root architecture and physiology in the field, where the conjunction of FSPMs and breeding approaches should ideally takes place. One way to address this challenge will be through technological progress, such as the development of underground radar techniques or transportable MRI apparatus which will allow for non-destructive *in situ* imaging of root structures and water fluxes in soil. Local physico-chemical properties of the soil could also be explored using underground sensor such as optodes which can currently be used to fine-map rhizotron or rhizoboxes but would need to be improved for field use. The acquisition of data regarding the physiological status of the different part of the root system within the soil is a more problematic issue that will require ingenious inventions in the domain of markers of physiological status. Another way to tackle this issue would be to develop integrated models coupling underground to aboveground functional processes. This would allow indirectly assessing the behavior of the underground parts of a plant through measurement of aboveground traits such as sap flow, stomata conductance or leaf-temperature, thus facilitating field-validation of model prediction.

We also mentioned the fact that while the rhizosphere was shown to play a critical role in the buffering of water stress during drought or submergence episodes (Carminati et al., 2010; Moradi et al., 2011), no current root model includes it (Dunbabin et al., 2013). As such, one of the challenges for root hydraulic FSPM is to integrate the rhizosphere layer as a dynamic interface between roots and soil. This presupposes additional investigation of the precise dynamics of the rhizosphere deposition and evolution throughout root development, and of its interaction with the soil processes. Depending on the complexity of the rhizosphere dynamics, its consideration might require either the extension of existing models or the development of completely new root FSPMs model paradigms.

More generally speaking, some of the main challenges for root FSPMs in the future are centered upon ecosystem integration. For example, it is known that soil microorganisms increase plant growth and tolerance to water stress and result in changes in root system morphology (Azcón-Aguilar et al., 1996), but no current root model take that into account. It would thus be interesting to consider the integration of biotic interactions in future root FSPM development. Of particular interest would be the investigation of the influence of mycorrhizae on water acquisition by plant roots. Of course, including this partnership in root FSPMs would necessitate being able to observe and quantify the dynamics of mycorrhizae development in soil, to estimate its impact on root developmental and physiological processes, and to measure water fluxes going from the soil to the plant through the fungi.

Regarding the topic of processes integration, current root hydraulic FSPMs can be used either to simulate water fluxes given static root architectures, or to simulate root growth under water acquisition-related developmental feedbacks. However, root development regulation does not only depend on soil water content and as such, root FSPMs need to evolve toward integration of the full range of regulatory processes impacting root growth and development, such as mechanistic description of nutrient perception and tropisms at the microscopic scale.

On the topic of scale integration, if root FSPMs are to be used as breeding tools, they will increasingly need to be able to integrate quantitative and qualitative knowledge from both extremities of the scale range. At the microscopic scale, root FSPMs will have to integrate rules for the genetic and hormonal regulation of root growth and physiology at the cellular level. For instance in the context of drought, it has been demonstrated that abscisic acid controls water stress tolerance mechanisms in later steps of root growth (Kholová et al., 2010). Other phytohormones such as auxin are also implicated in the complex feedback systems of root developmental regulation and environmental perception (Lavenus et al., 2013). Several models of mechanistic regulation by auxin of the different steps of root branching (initiation and emergence) have already been proposed (Lucas et al., 2008; Péret et al., 2012, 2013). The processes described in those models rely essentially on the dynamical reorientation of auxin fluxes at the cellular level by changes in auxin transporters expression and localization. In the particular case of root emergence, there is a clear link between cellular hydraulics and the auxin regulatory processes (Péret et al., 2012). This hint at further coupling mechanisms that will need to be explored to explicitly link the hydraulic state of the whole root system to its cellular development and resulting architecture. While one may argue that considering microscopic scale processes would unnecessarily complicate the FSPMs, the identification of explicit morphogenetic mechanisms at the cellular level would be of tremendous help to link FSPMs prediction with genetic studies such as QTL or GWAS analysis. The culmination of this would be the possibility to model the impact of changes at the level of a single component of the genetic network on the development and physiology at the root system scale.

On the other side of the scale range, at the macroscopic level, two more specific challenges remain. First, root FSPMs will need at some point in time to be connected to shoots FSPMs. While it is easier to consider roots and shoots independently, there is a necessary developmental coordination between aerial and underground plant organs. Some simple models connecting shoot and root already exists (Sperry et al., 2016) and will need to be expended upon so that both roots and shoots FSPMs can benefit from the creation of unified virtual plants models. Second, root FSPMs are mainly used to consider a single plant in interaction with its environment. In the context of breeding and agricultural production, a plant being virtually alone in its environment is an exceptionally rare case. Root FSPMs will thus need to be adapted to investigate crop-like situation including inter-individual competition and/or cooperation. It will also be interesting to use multiple parallel distinct root FSPM to study inter-specific interactions and their potential impact on agricultural practices such as inter-cropping.

In the end, the future of functional-structural plant models represents both an incredible opportunity and an incredible source of technical and intellectual challenges, which will require scientific cooperation through fields far out-reaching plant biology. While it remains to be seen whether the Second Green Revolution will actually precede or follow the advent of true virtual plants successfully integrating all biological scales, one thing is for certain: virtual plants have taken root.

## Author contributions

AN, VV, CP, and ML all contributed to the redaction of this review.

### Conflict of interest statement

The authors declare that the research was conducted in the absence of any commercial or financial relationships that could be construed as a potential conflict of interest.
